# How Public Health Organizational Structure Affected the Response to the COVID-19 Pandemic: A Case Study in British Columbia, Canada

**DOI:** 10.3389/ijph.2024.1606638

**Published:** 2024-01-23

**Authors:** Peter Berman, Michael Cheng, Elvira Bridget, Laura Jane Brubacher, Candice Ruck

**Affiliations:** ^1^ School of Population and Public Health, University of British Columbia, Vancouver, BC, Canada; ^2^ School of Public Health Sciences, University of Waterloo, Waterloo, ON, Canada

**Keywords:** public health, policy making, COVID-19, institutions, governance, organization

## Abstract

**Objectives:** This study sought to examine how public health organizational structures affected decision-making and provides recommendations to strengthen future public health crisis preparedness.

**Methods:** The Institutions-Politics-Organizations-Governance (IPOG) framework and an organizational lens was applied to the analysis of COVID-19 governance within British Columbia (BC). Organizational charts detailing the structure of public health systems were compiled using available data and supplemented with data collected through key informant interviews.

**Results:** In response to the COVID-19 pandemic, BC initiated several changes in its public health organization. BC’s COVID-19 response attempted to utilize a centralized command structure within a decentralized health system. Four key themes were identified pertaining to the 1) locus of decision-making and action; 2) role of emergency structures; 3) challenges in organizational structure; and 4) balance between authority and participation in decision-making.

**Conclusion:** The organizational adaptations enabled a substantively effective response. However, our findings also illustrate deficiencies in organizational structure in the current public health system. Two recommendations for consideration are: 1) a more formal vertical organizational structure; and 2) developing new mechanisms to link health and general emergency response structures.

## Introduction

The COVID-19 pandemic has presented an unprecedented challenge to the structure and function of health systems. Public health systems in particular have been at the forefront of the pandemic response and, as such, have come under intense scrutiny during the response to COVID-19. In some jurisdictions, public health organizations responded effectively to the pandemic, while in others they were diminished or limited in their roles [1].

The organization of public health systems varies significantly between countries as well as across jurisdictions within federal countries. Such is the case in Canada, where the provinces retain significant autonomy over healthcare and thus display notable diversity in organization of public health systems. These structural variations may partially explain the distinct differences that were observed in jurisdictional responses to COVID-19.

In the province of British Columbia (BC), the organizational structures constituting the public health system evolved in accordance with the pandemic’s progression [2]. Some of the direction for this organizational re-configuration came from BC’s Pandemic Provincial Coordination Plan, which was modified at the outset of COVID-19 in February 2020 [3]. Despite this prior preparedness, considerable ambiguities arose during the COVID-19 pandemic regarding changes in organizational structure, roles and responsibilities [4].

This study aims to describe the organizational structures involved in BC’s public health emergency response for COVID-19. Additionally, we will: 1) examine how these organizational structures affected decision-making; and 2) suggest how future preparedness for public health crises might be strengthened by addressing key challenges identified in our findings.

## Methods

### Research Context and IPOG Framework

This research is part of a broader, in-depth case study within BC that applied a mixed-methods conceptual framework to investigate the influence of institutional (I), political (P), organizational (O), and governance (G) factors on the COVID-19 response, the details of which have been published elsewhere [5, 6]. This framework enables clearer distinction between concepts of “institutions” and “organizations” which are often used as synonyms in public administration literature. It also focuses on the decision-making processes as “governance”, a term which often is not well-defined. Policy decisions to address the pandemic are framed as occurring at the interface of politics and organization, influenced by broader contextual factors and institutions as accepted norms and rules of social behavior.

The study described here applies this IPOG framework to the analysis of COVID-19 governance within BC. Ethics approval for this research was provided by the University of British Columbia Research Ethics Board (ID#H20-02136).

### What Is the “Public Health System”?

There is no single universally-accepted definition of the “public health system” with which to frame the scope of our analysis. This is an important topic for future investigation. For the purposes of this research, we focused on key organizations in BC that are specifically identified as having public health functions, as well as on the parts of other organizations with wider responsibilities that are charged primarily with managing public health functions. We also focus particularly on those organizations urgently needed during the COVID-19 crisis.

### Data Collection

#### Key Informant Interviews

From July 2021 to January 2022, 18 semi-structured, key informant interviews were conducted with stakeholders in British Columbia’s COVID-19 response, including elected officials, Ministry of Health employees, public health officials, actors from BC research institutes, labour union representatives, and other relevant provincial agencies (Table 1).

**TABLE 1:** Role/positions of key informants interviewed for this study (Institutions, Politics, Organizations, and Governance in the COVID-19 Response. British Columbia, Canada, 2020).

Role/Position	Participant ID
1. Provincial-level health officials	IDI1, IDI2, IDI3, IDI4, IDI5, IDI7, IDI8^a^, IDI9, IDI12, IDI13^a^, IDI16
2. Regional-level health officials	IDI6, IDI10
3. Elected officials	IDI15
4. Civil society actors (e.g., non-profit research organizations, unions)	IDI11, IDI14, IDI17, IDI18

^a^ Former role (retired).

Respondents were selected based on their involvement in the COVID-19 response and to ensure diverse representation of organizational roles and responsibilities. Snowball sampling was utilized to identify additional respondents with relevant backgrounds. All respondents were invited to participate via email and provided written informed consent. Interviews were conducted virtually over Zoom^®^ with the exception of one interview conducted in a hybrid virtual/in-person format (duration of all interviews = approximately 60 min). Interviews focused on decision-making processes and actors involved in BC’s COVID-19 response from January to December 2020. Following each interview, research team members engaged in peer debriefing and discussed observations that were integrated into the analysis. Interviews were audio-recorded via Zoom^®^ software, with permission, and transcribed using *NVivo© Release 1.5* software. Each transcript was manually verified for accuracy by two research team members.

#### Organogram Development

Organizational diagrams (organograms) were developed based on the World Health Organization’s “organigraph” approach [7], illustrating configuration of and linkages across structures at different levels of public administration [5]. Public documents, websites, and key informant interviews were used to identify organizational units and sub-units, their positioning in relation to one another, and constituent relationships of accountability/reporting. Some structures and relationships were less well documented than others, resulting in some uncertainties [8]. During organogram development, some of these gaps in understanding were addressed in key informant interviews with participants knowledgeable in public health organizational structure and function in BC.

### Data Analysis

An inductive thematic analysis using a constant comparative method was conducted to analyze interview data [9]. Specifically, initial open coding was used to generate a list of potential codes and preliminary themes. Framework analysis was also employed as a visual tool to generate analytic insights across stakeholder perspectives and, in particular, to identify points of convergence and divergence across perspectives [10]. These insights were integrated into ongoing coding of the data, involving merging, re-organizing, and consolidating codes to create a parsimonious codebook to fit the data [11].

To provide some examples, interviews were coded to identify content related to the following high-level themes: historical context, identification of specific organizations and roles, specific COVID-19 related structures created, organizational operations, interviewees perceptions of organizational roles, organizational independence, organizational changes during the pandemic, hierarchical factors in organizational response, differences between formal structure and actual decision-making, informal structures, coordination between organizations, and clarity-complexity- integration specific recommendations on organization.

*NVivo©* software was used for organization of codes and subsequent coding and retrieval of interview excerpts. Triangulation of data across participants and analytic methods (e.g., interviews and organogram development), collaboration among team members in reviewing and revising ongoing analysis, and the creation of a data audit trail were procedures that contributed to data validity [12].

The organogram visually identified relationships between organizations and actors, and pathways of reporting and accountability, which complemented and expanded our understanding of themes developed through the qualitative analysis.

## Results

*“The province has a huge opportunity to be thinking about how you actually make change in complex systems...not just default[ing] to pretty structured ways of working, but adapting to a changing context.”*—Research Stakeholder

### Public Health System Organizational Structures in BC Before and During the COVID-19 Pandemic

#### Pre-COVID-19 Public Health Organization

**FIGURE 1:**
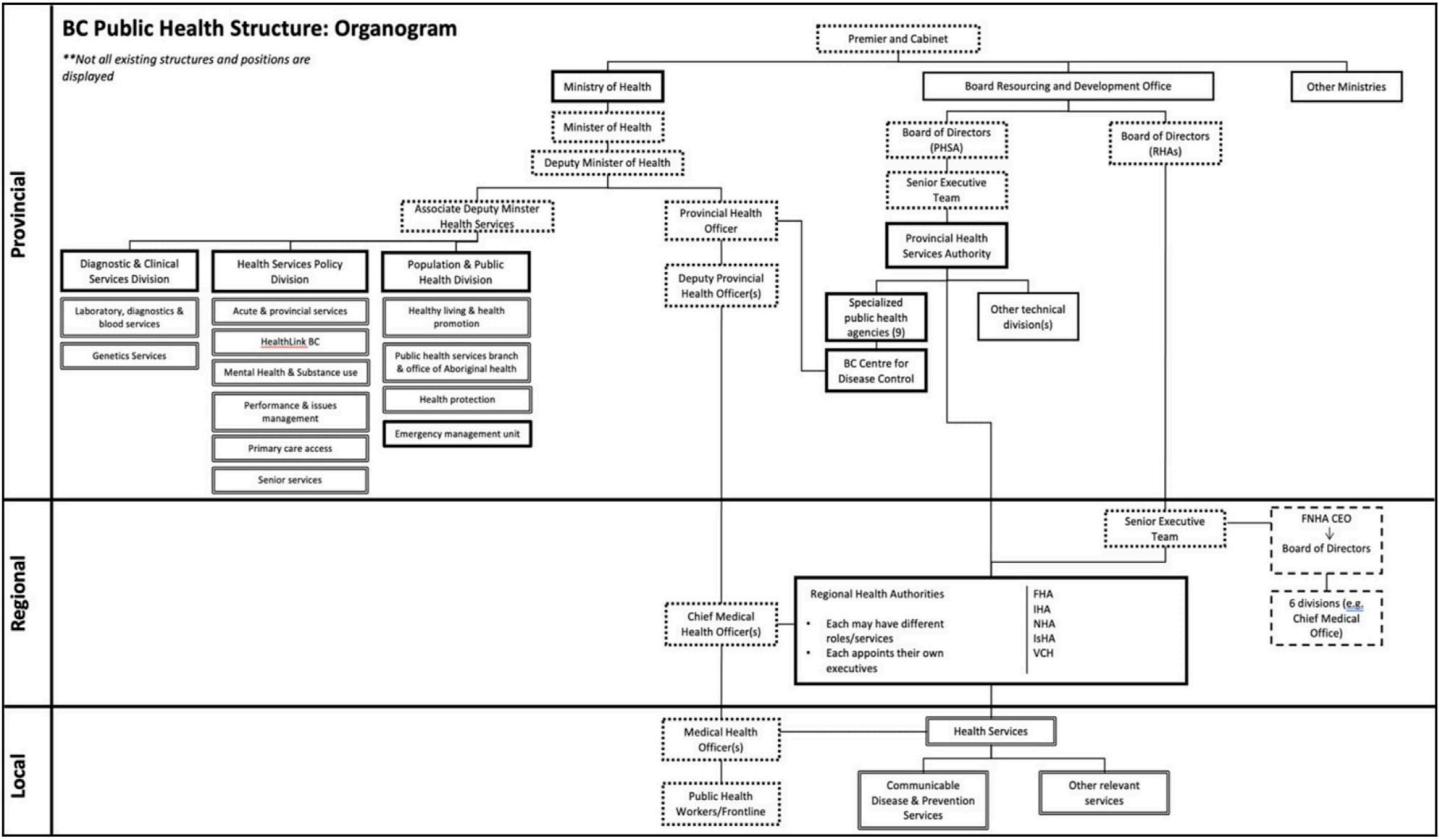
Organogram depicting the public health system according to provincial-regional-local levels of government prior to COVID-19 (Institutions, Politics, Organizations, and Governance in the COVID-19 Response. British Columbia, Canada, 2020). Solid borders = health organizations; dotted borders = positions/roles; solid connecting lines = hierarchy. Additional detail about other organizations represented here and their denotation are referenced elsewhere [8].

Respondents discussed key “pre-COVID-19” organizations and roles in relation to the COVID-19 response, and described both their responsibilities and function within BC’s public health system, as well as the reporting or other working relationships among them (Figure 1).

Under British Columbia’s *Public Health Act* [13], the Provincial Health Officer (PHO) is the senior public health physician responsible for monitoring public health needs, advising the Ministry of Health (MoH), setting standards of practice, and monitoring the performance of regional and local medical health officers (MHOs). The PHO position is created by an order from the lieutenant governor as representative of the sovereign (order-in-council). It is a senior civil service position. The PHO can also declare a provincial public health emergency under the *Public Health Act* which confers legislative authority to make particular decisions, and is required to advise the government.

The British Columbia Centres for Disease Control (BCCDC) is considered the technical and scientific body of the provincial public health services that functions in supporting the provincial public health functions. Despite having statutory decision-making authority, the PHO does not have formal hierarchical authority over BCCDC, which is organized under the Provincial Health Services Authority (PHSA), a semi-autonomous provincial agency that embeds multiple health organizations. The PHO also does not have formal authority over the Chief MHOs and MHOs at the regional and local levels, who are the key personnel managing public health activities on the ground and are formally employed by the sub-provincial agencies.

The Regional Health Authorities (RHAs) are government agencies charged with administering healthcare delivery in five geographic regions, supplemented by some clinical functions administered by the PHSA. In addition, the First Nations Health Authority (FNHA) has specific separate roles in relation to First Nations populations. The primary role of the RHAs is clinical services administration with an emphasis on hospital-based services. CMHOs and MHOs are staff of the RHAs, primarily charged with carrying out specific public health-related activities.

#### Changes in Organizational Structure in Response to COVID-19 During 2020

**FIGURE 2:**
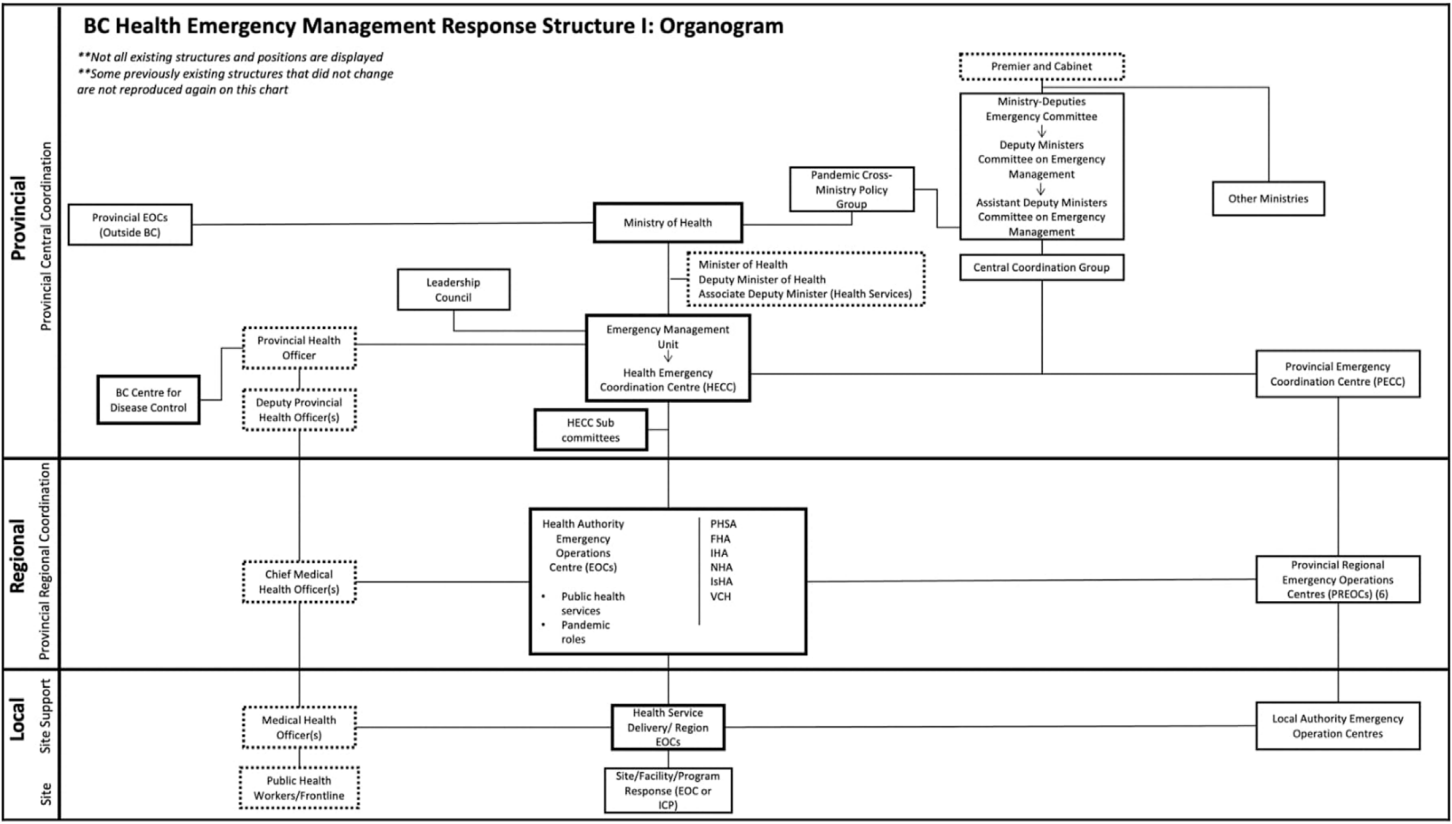
Organogram depicting the public health system according to provincial-regional-local levels of government (Institutions, Politics, Organizations, and Governance in the COVID-19 Response. British Columbia, Canada, 2020). Solid borders = health organizations; dotted borders = positions/roles; solid connecting lines = hierarchy. Additional detail about other organizations represented here and their denotation are referenced elsewhere [8].

In responding to the acute crisis of COVID-19 during 2020, BC instituted several waves of changes in public health organization [14]. The initial changes occurred during the first half of 2020 following declarations of Health and Provincial States of Emergency (Figure 2). At the higher levels, ministry-level committees on emergency management and policy were activated. Two concurrent emergency “coordination centres”—a Health Emergency Coordination Centre (HECC) and a Provincial Emergency Coordination Centre (PECC), were also activated in response to the States of Emergency. The former was under the Ministry of Health, under the leadership of the PHO and a Deputy Minister of Health. The latter was under the Ministry of Public Safety.

The HECC and the PECC also coordinated a number of *ad hoc* committees and coordination groups with large and sometimes shifting membership. Since the meetings of these groups were conducted almost entirely virtually, it was difficult to ascertain who participated personally in frequent online meetings and briefings.

Regional Emergency Operations Centres were also created within both the health (Health Authority Emergency Operations Centres) and provincial emergency structures (Provincial Regional Emergency Operations Centres). It is worth noting that the geographic delineation of regions for health and general emergencies are not identical.

**FIGURE 3:**
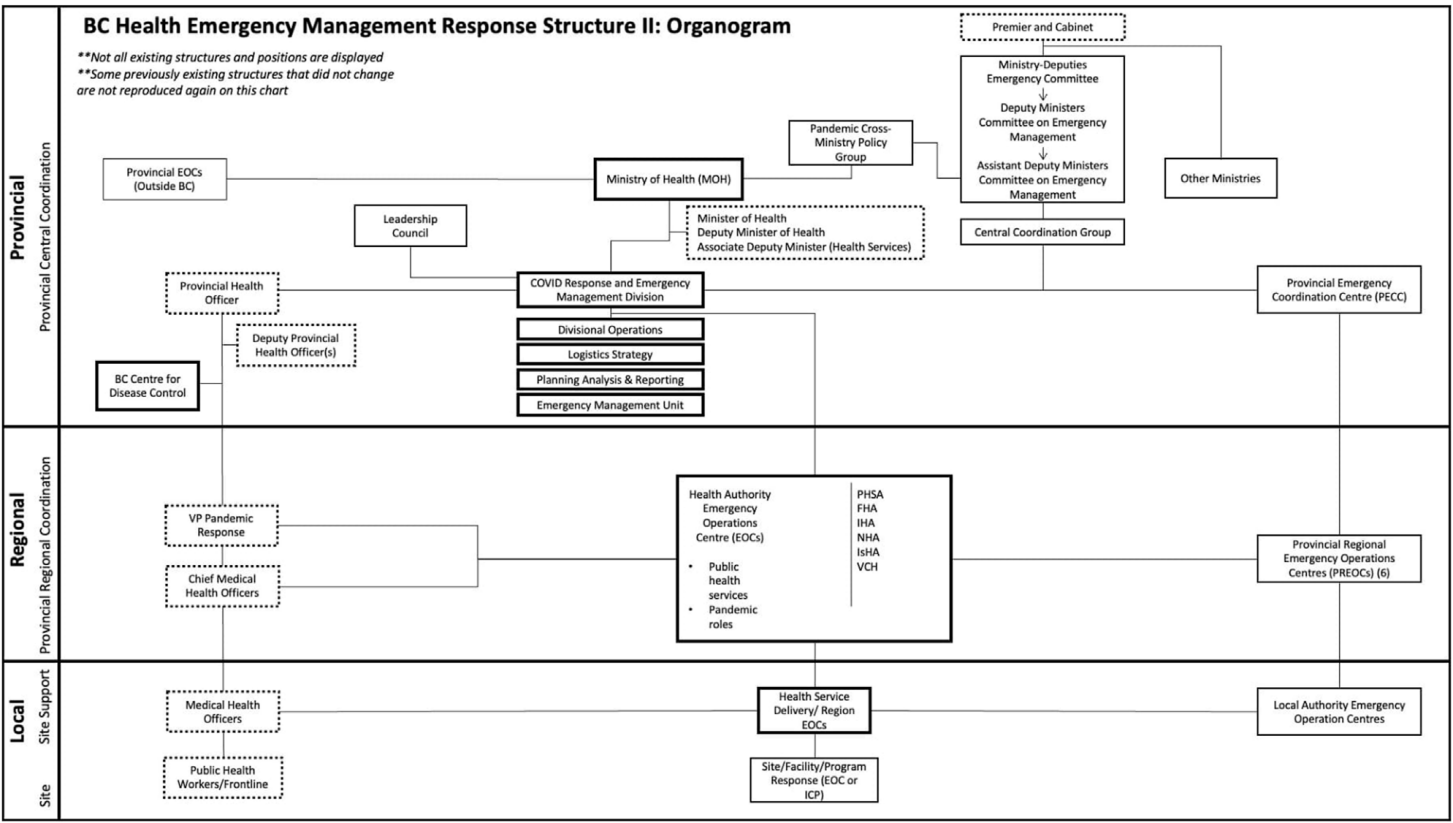
Organogram depicting the public health system according to provincial-regional-local levels of government (Institutions, Politics, Organizations, and Governance in the COVID-19 Response. British Columbia, Canada, 2020). Solid borders = health organizations; dotted borders = positions/roles; solid connecting lines = hierarchy. Additional detail about other organizations represented here and their denotation are referenced elsewhere [8].

As the first wave of infections subsided by mid-2020, there was a shift from an emergency management structure to a more routine operations structure (Figure 3). Management of the pandemic response was assigned to an MoH Deputy Assistant Minister. The RHAs appointed Vice Presidents of Pandemic Response. In most cases, these positions were not given to the Chief MHOs in the RHAs but were held separately. More details on these changes have been published elsewhere [7].

The Health and Provincial States of Emergency remained in effect during this period and into 2021; thus, the PHO and the Minister of Public Safety retained extraordinary powers to issue public health and safety orders, such as the closure and occupancy of public spaces, and vaccination roll-outs and mandates.

### How Public Health Organizational Structures Influenced COVID-19 Decision-Making and Response

The organizational response in BC was vigorous, timely, and complex. From an organizational perspective, the COVID-19 response in BC attempted to overlay a centralized command structure on a decentralized health system. Respondents described their perceptions of the resulting organizational dynamics.

#### Locus of Decision-Making and Action: Provincial Versus Regional Structures

As noted above, the province-level public health organizations do not have line authority over the RHAs or the public health personnel within them. Respondents described experiencing difficulties with the locus of decision-making in the COVID-19 response, whether at regional or provincial levels. For instance, many discussed the independent actions of RHA structures, particularly in the early COVID-19 response (March to August 2020). As one respondent noted, *“the various elements [RHAs] don’t all work well together. We were all doing something and didn’t want to rely on the PHSA or MoH...rightly or wrongly, we were working independently”* (IDI6). This was largely attributed to the level of urgency required in the response and a sense that provincial structures (e.g., PHSA or MoH) were lagging in providing guidance or *“two steps behind”* (IDI6). As another regional actor shared, *“there was always that period where we just had to do some things. We had to make that decision. And then very often the sort of provincial consensus or direction came a bit afterwards, after the fact”* (IDI10). RHAs reported being consumed with local, immediate outbreak management and, thus, initially looked “across” the organization to other regional structures, rather than “up” to provincial structures for input on guidelines, policies, procedures, and management techniques.

Other respondents also spoke to independent functions of the regional structures and associated challenges, particularly in a health emergency context when a more coordinated response might be necessary. For instance, when asked to position them on an organizational map, a provincial actor placed PHSA *“at the same level of the other [regional health authorities]”* providing *“horizontal support”* and went on to say that this positioning *“sometimes created friction because certain health authorities wanted to do something in a certain way, but there was a need for a provincial response”* (IDI5). This independent dynamic was echoed by another provincial actor working closely with regional structures, who perceived that *“all of the health authorities seem to be independent, do what they believe is appropriate and follow the advice, or more of an advisory capacity, the information coming from [the PHO] and more provincial central body rather than taking that as marching orders, if you will”* (IDI17).

Despite evident regional independence in certain functions and in the ability to act, both regional and provincial actors acknowledged the benefits of some centralized and coordinated decision-making and that, for certain points in the pandemic, regional actors were largely becoming accustomed to more provincial leadership, most notably around the widespread implementation of vaccine roll-out. As a provincial actor shared, in relation to vaccination:

*There [was] a lot of discussion that took place and in the end, there need[ed] to be a decision. Single site orders at the outset when everything started. That was a critical decision that was driven down by [the] top, down by [the PHO]. And we were all on board. But the MHOs were raising questions as to what this means. But then they rallied* (IDI5).

It was provincial actors who more often perceived, however, the importance of a provincial, centralized approach to decision-making more broadly. As one actor stated, *“I do think there is a time and place for regional decisions and there is a time and place for an overall provincial decision,”* and further noted the importance of a centralized locus of authority in emergency situations when “*someone [has] to call the shots”* (IDI7). Similarly, another observed that a provincial approach was *“not only necessary, but yields a lot of good for the system”* (IDI5), while another provincial actor attributed the success of BC’s response, in part, to:

*[The PHO’s] ability to keep a consistent message at the provincial level, despite how difficult it was and upsetting it was for different layers within the public health system to hear something that didn’t necessarily align with what was happening in their local area* (IDI16).

Overall, one actor’s statement seems to reflect the general perceptions of the provincial-regional relationship, that it “*sometimes worked very well and sometimes was challenged by the MHOs of the RHAs”* (IDI5). This friction was reiterated by a provincial actor who, nonetheless, emphasized the importance of centralized decision-making in a crisis:

*We have a small group of public health officials who are willing to make decisions. They don’t always agree. They almost never agree. So, at the end of the day, when something like this is happening, it does end up having to be [the PHO] saying, “okay this is what we’re doing, right?”* (IDI16).

#### Role of Parallel, Concurrent Emergency Structures in COVID-19 Response

The activation of emergency structures, according to the incident command structure, was designed to facilitate centralization of authority and action in the early COVID-19 pandemic response (March to August 2020). According to respondents, this made for streamlined and efficient decision-making largely related to logistics and operations. In the words of one actor, emergency structures, specifically the Health Emergency Coordination Centre (HECC), *“does provide a very clear touchpoint for the health authorities and more active coordination, perhaps”* (IDI12). However, the effectiveness of this emergency structure was also questioned, that *“when things are very operational, sometimes the HECC isn’t really the right structure. And we’re actually thinking there might need to be more like a PHSA operation centre that provides support to the [regional] health authorities for this”* (IDI12). The existence of two parallel emergency response structures under different ministries also required additional efforts to coordinate the dissemination and implementation of various orders to control infection, such as those relating to commercial spaces.

Beyond the first 6 months of acute emergency response, respondents identified an adaptation phase for the remainder of 2020 (September to December). This was largely characterized by organizational re-configuration (Figures 2, 3), the rationale for which was described by a Ministry of Health actor:

*The HECC structure is really good at getting things done very, very quickly. But it does that by centralizing authority and it centralizes authority away from where it should be in government, which is with the Ministry and the government. So in May and June, there were a series of discussions in the Ministry and I think within government saying that actually we needed to, and I think the words we used were, “bring the COVID response back into the framework of government” so that it became a program, it became something that the Ministry of Health did and what the health system did and what government did. As opposed to something, a separate structure, that was set up to respond to this very urgent thing* (IDI3).

#### Perspectives on Challenges in Organizational Structure: Implications for COVID-19 Governance

The organizational complexity depicted within the public health system was identified as a challenge to effective and efficient action. One provincial actor shared that:

*The Medical Health Officers (MHOs) within the regional health authorities report to the Regional Health Authorities, but have a link, have a connection, have a relationship with [the PHO], but they don’t report to [the PHO]. So when [the PHO] makes a call as to whether or not [to] go left, right, or centre, they have to negotiate that decision with the MHOs. And that sometimes creates complexity or sometimes a lag in terms of being able to act or a discrepancy in terms of response per regional authority. So that’s important* (IDI5).

Similarly, an actor from a provincial organization outside of public health translated this complexity into a lack of *“ability to be nimble, in the ability to execute, in the ability to take something in [e.g., evidence] and truly make it practical, realistic, and understandable and then implement it”* (IDI17). Relatedly, multiple respondents referenced a lack of clarity in public health organizational structures, in terms of hierarchy/reporting and also a formal process for decision-making via these structures. In the context of health crisis response, a provincial actor noted that *“We haven’t defined our roles and responsibilities well. We just sort of fell into them*.*”* (IDI7).

Some respondents also shared challenges associated with the positioning of particular sub-provincial organizational structures in relation to one another and had recommendations for more cohesive functioning. Many perspectives related to the position and function of the BCCDC relative to PHSA. As one actor shared, *“there was a time when [BCCDC] was its own agency, but it got sucked into PHSA and PHSA is just like, okay. And all of the agencies struggle with understanding what is going on”* (IDI16). Despite the current formal positioning of BCCDC within PHSA, there existed an evident distinction in their respective functions, with BCCDC being responsible for the public health response and PHSA for the health system response. This relationship between BCCDC and PHSA was further highlighted by another respondent:

*BCCDC says it’s part of PHSA, it’s an arm of PHSA, reporting through the hierarchy and all the way up to the board. That’s the way it is. In reality, it’s not. The BCCDC should be a central function that would be an overarching function of the regional health authorities and the MHOs of the health authorities should be part of the BCCDC planning and operations and take part of that and buy into it. It’s not the case necessarily. It’s negotiations, negotiations. And sometimes it’s also a little bit of tug of war...the BCCDC needs to operate in that environment and be able to influence* (IDI5).

Another challenge reported by some respondents was a perceived gap between the issuance of public health orders and the capacity to formulate the specific regulations implied by those orders and to implement them. One senior official noted that while the PHO had the legal authority to issue orders related to public spaces or occupancy limits, neither the PHOs office nor the RHAs had the capacity to implement those orders in practice nor to regulate and enforce them. The absence of a vertically-organized public health implementation structure meant that this role fell to other, non-health organizations. According to one respondent, this dynamic meant that *“public health and [BC]CDC are less agile with respect to really implementing what they would like to implement”* (IDI17).

#### Balance Between Authority and Participation in COVID-19 Decision-Making

Descriptions of the organizational structures of the public health sector depicted complexity: an abundance of committees, councils, actors, and meetings involved in coordinating the COVID-19 response. Respondent views differed on whether this was largely successful in creating a sense of an informed, engaged, collective response to the crisis or instead created confusion.

Many noted a more implicit, centralized decision-making process, often reported to involve a small number of actors primarily including the PHO, Deputy Minister of Health, and Minister of Health, and the Premier in decisions affecting multiple sectors. One actor, however, noted that decisions were made overall *“either top down from government or leadership council. And sometimes, often times, it was a little bit of both. ‘This is where we’re going with this. What do you think?’”* (IDI5). As expressed by multiple actors, the hallmark approach of public health involves this “*influencing, [which] is big in the healthcare system, much more than top down decision-making”* (IDI5). There reportedly exists much negotiation among public health officials, with *“decision making [being] in the hands of everyone and no one in particular...whenever you want to bring the health system together, it’s like herding cats”* (IDI5).

Despite these complexities and disparate viewpoints, the overall consensus in BC was that the organizational processes were successful during COVID-19. As one actor emphasized, the public health organizational structures *“sound very complex and a little bit chaotic, but it works in the end, right? It works because the BC response was pretty good. Is there tweaking that would be useful? I personally think so, yes. There’s lots of cooks in the kitchen, that’s for sure”* (IDI5).

## Discussion

In a Canada-wide review, British Columbia’s early responses to the COVID-19 pandemic have been described as a “local success” in reducing the health impact of the initial wave of infection although less so in later waves [15]. Reviews of the development and use of research engaging a variety of actors has been cited as an important contributor to positive results, although much of this relates to clinical responses [16]. The internal review commissioned by the provincial government reported an overall creative and flexible organizational adaptation to a challenging and rapidly changing situation but noted that structural factors could be improved in the future [4]. While relatively successful, the need for *ad hoc* adaptations to the rapidly evolving crisis can also be seen as illustrating some deficiencies in the organizational structure of the current public health system. Based on our review, we recommend the following be given further consideration:

• A more formal vertical organizational structure to identify priority population health needs and action strategies and lead implementation. In BC, public health organizations are distributed across a variety of separate vertical structures. There are few formal lines of authority or planning and management linking these distributed organizations. As British Columbia’s response and some of our interviewees observed, most of the formally-appointed public health actors in the system (e.g., MHOs) are linked to higher-level strategies and priorities by informal networks and *ad hoc* mechanisms. Respondents noted the challenges of depending on informal networking. So we ask, would BC be better served by a more formal vertically-integrated structure for population health? Or perhaps even a separate Ministry of Population Health? This might also provide a mechanism for better response to other emerging public health challenges, as noted below.
• Developing new mechanisms to link health emergency response with general emergency response structures. One clear lesson emerging from the evolution of the COVID-19 pandemic was that health emergency response organizations needed to rely on non-health organizations to implement the orders which the PHO is legally empowered to declare. In BC’s COVID-19 response, *ad hoc* mechanisms were needed to bridge these connections and sometimes they had mixed results in terms of communication, timing of action, and effectiveness. The somewhat different geographic areas delineated as RHAs and general emergency response regions also illustrate some level of disconnect. Would it be feasible to plan in advance for a more integrated emergency response organizational structure that could be triggered by a Provincial State of Emergency?

We propose that this case study approach has potentially much wider relevance to the future of public health action. Specifically, the concept of a “public health system” is often used to describe a very broad frame of action encompassing many organizations only some of which are specifically public health organizations. Policymakers may need a more practical operational definition of the key public health actors to focus the provision of key inputs such as funding and human resources and to be able to assess performance. In addition, future population health crises will not only be those related to infectious disease, but also mental health, managing multi-morbidities, and mitigating the negative impacts of commercial determinants of ill-health. Public health organizations will need stronger links to other actors in emergency response as well as explicit roles in preventing increasingly costly medical conditions. These needs are already apparent in British Columbia. More explicit organizational analyses are needed in many other jurisdictions to strengthen population and public health impacts.

This study had some limitations. We only interviewed a relatively small number of actors in a large and complex public system. Interviews were conducted between July 2021 and January 2022. Those interviewed later had a different perspective, having observed the pandemic progression longer than those interviewed earlier. To address this, we kept the interview guide largely consistent and focused on particular events and COVID-19 “waves” that all interviewees could discuss from similar temporal perspectives. We regret any errors in interpretation and analysis for which we the authors are solely responsible.

This study reveals some of the ways in which the organizational structure of the public health system in BC hampered the efficiency of the pandemic response. BC, like other jurisdictions, now has an opportunity to apply the lessons learned from COVID-19 to be better prepared for the next health emergency, including at the organizational level.
